# Collected Papers on Circulation and Respiration

**Published:** 1907-06

**Authors:** 


**Collected Papers on Circulation and Respiration.** By Sir LAUDER BRUNTON, M.D. First Series. Experimental. Pp. xiii., 696. London: Macmillan & Co., Limited. 1906.

There is no need for us to draw attention to the very valuable experimental work which has been carried out by Sir Lauder Brunton on problems connected with circulation and respiration. In this volume is collected a series of papers which was published by him between 1867 and 1883. These papers are exceedingly interesting, and there will doubtless be many who will be glad to have them all in one volume.

